# Using Vesicular Stomatitis Virus as a Platform for Directed Protease Evolution

**DOI:** 10.1002/cpz1.70074

**Published:** 2024-12-23

**Authors:** Francesco Costacurta, Stefanie Rauch, Dorothee von Laer, Emmanuel Heilmann

**Affiliations:** ^1^ Institute of Virology Medical University of Innsbruck Innsbruck Austria; ^2^ Biological and Environmental Science and Engineering Division KAUST Thuwal Saudi Arabia; ^3^ These authors contributed equally to this work

**Keywords:** coronaviruses, evolution, M^pro^, protease inhibitors, resistance, SARS‐CoV‐2, vesicular stomatitis virus

## Abstract

Antiviral drugs are essential medications to save the lives of infected people. However, they are under constant threat to become ineffective as viruses evolve quickly. Studying the development of resistance is therefore paramount to understand the impact of mutations on pharmacological treatment and to make informed decisions. Yet, such studies are open to scrutiny, as they are considered gain‐of‐function research, which is especially problematic with viruses of pandemic potential. In this article, we present a protocol that allows for the selection of antiviral resistance mutations safely, without using the actual virus (e.g., SARS‐CoV‐2, MERS‐CoV). Instead, we use vesicular stomatitis virus (VSV) that serves as a surrogate virus; like other RNA viruses, it is prone to mutations due to its polymerase lacking proofreading. By replacing parts of the VSV genome with transgenes from other viruses, VSV becomes dependent on their function. Thus, we can mount a selection pressure with antivirals targeting the transgenes to subsequently sequence selected resistance mutations. This article provides a protocol for this process as well as a sequencing pipeline that we used to collect mutations. © 2024 The Author(s). Current Protocols published by Wiley Periodicals LLC.

**Basic Protocol**: Using VSV as a platform for directed protease evolution

**Alternate Protocol**: Dose response assay with TCID_50_ readout

**Support Protocol 1**: A pipeline for high‐throughput VSV sequencing

**Support Protocol 2**: Rescue of VSV

## INTRODUCTION

**Figure 1:**
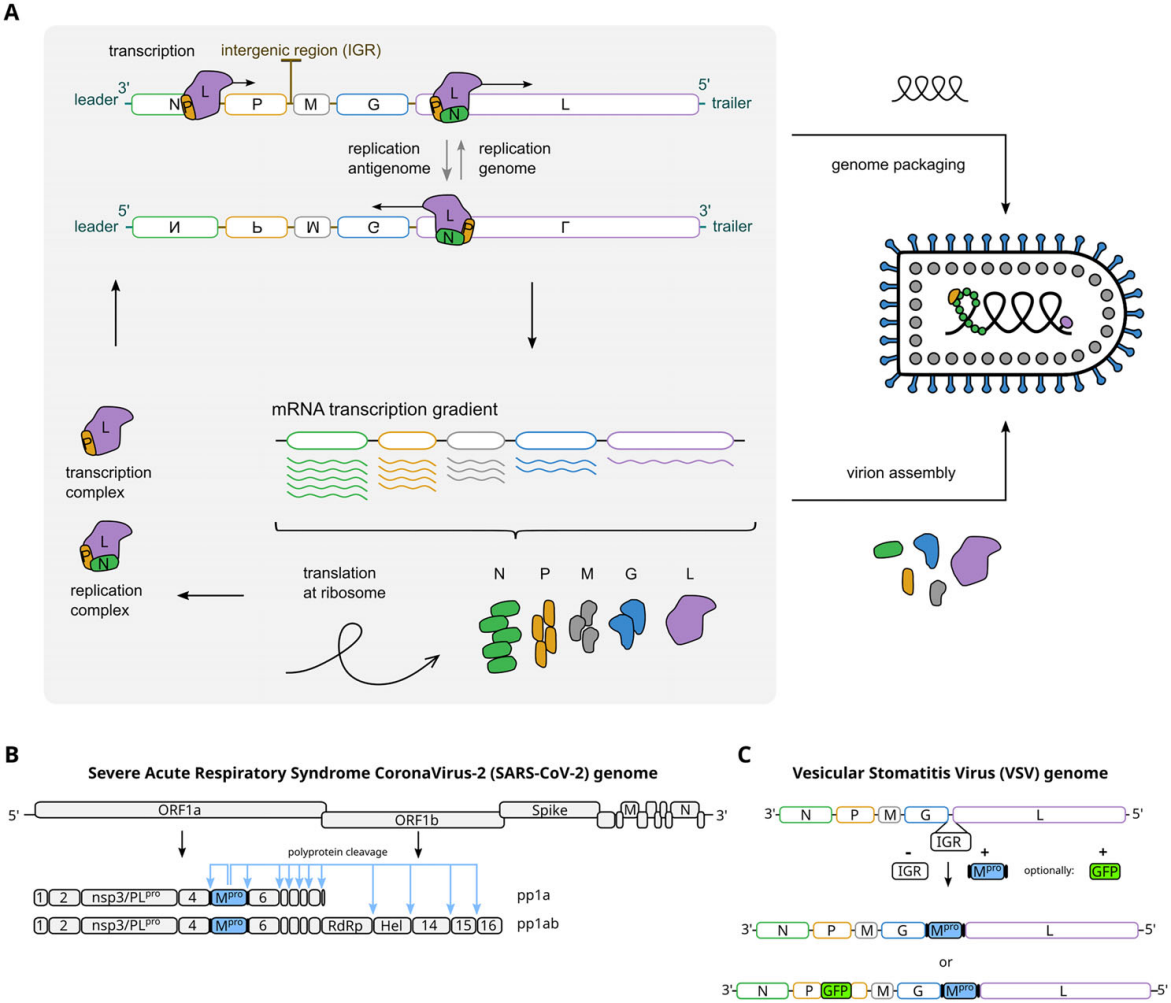
VSV, SARS‐CoV‐2, and chimeric VSV‐M^pro^. (**A**) VSV mRNA transcription is carried out by P‐L complexes. The transcription complex is guided by the leader and trailer sequences, as well as the intergenic regions (IGRs). The IGR sequences mediate an ∼30% L drop‐off at every gene junction, leading to a transcription gradient from 3′ (N) to 5′ (L). The mRNAs are then translated. The mRNA gradient is reflected by the ratio of the five VSV proteins, where N is the most abundant and L the least. (**B**) The SARS‐CoV‐2 genome translates into two large polyproteins at the ribosome directly from its positive‐sense, single‐stranded RNA genome. The polyproteins pp1a and pp1ab are then processed by two proteases (PL^pro^ and M^pro^). The main protease (M^pro^) cleaves 11 sites within pp1a and pp1ab, thereby producing functional proteins. (**C**) Replacing the function of an intergenic region with that of M^pro^ results in VSV‐M^pro^, a virus relying on M^pro^ for its replication. Adding a monomeric GFP version as intramolecular insertion into P facilitates viral readouts. Panel A was adapted with permission from Rauch et al. (2024).

Vesicular stomatitis virus (VSV) is a virus that belongs to the *Rhabdoviridae* family, and it is widely studied to understand related, non‐segmented negative‐sense RNA genome viruses, such as Ebola, Nipa, and Rabies (Pringle & Easton, 1997). In contrast to these related viruses, VSV is mostly harmless to humans, requiring only biosafety level 2 (BSL‐2) laboratories. The genome comprises five proteins, spanning from 3′ to 5′: nucleoprotein (N), phosphoprotein (P), matrix protein (M), glycoprotein (G), and the large protein/polymerase (L). These viral genes are transcribed sequentially by the VSV polymerase in complex with the cofactor P. The regions of the genome that are located in between these five genes are called intergenic regions (IGRs) and are responsible for separate gene transcription. Furthermore, they guide the polymerase in a start‐stop mechanism that leads to a decreasing transcription gradient of messenger RNAs from N to L (Abraham & Banerjee, 1973; Ball & White, 1976). This transcription gradient is then reflected in the amount of protein translated at the ribosome, where N is most and L least abundant (Fig. 1A). The transcription strategy of VSV is therefore that of producing sub‐genomic RNAs, i.e., mRNAs.

Other RNA viruses use different or partly overlapping strategies. Coronaviruses, for example, also produce sub‐genomic RNAs as mRNAs. However, in addition, two thirds of their positive‐sense RNA genome are directly translated at the ribosome into two large polyproteins, pp1a and pp1ab. These two polyproteins are then processed by the Papain‐like protease (PL^pro^) and the main protease (M^pro^), which are therefore responsible for the generation of separate, functional, viral proteins. The severe acute respiratory syndrome coronavirus 2 (SARS‐CoV‐2) is part of the *Coronaviridae* family and, due to the recent pandemic, is its most well‐known member (V'kovski et al., 2021) (Fig. 1B).

The aim of the protocols described here is to avoid gain‐of‐function research with a pandemic pathogen while facilitating main protease (M^pro^) inhibitor resistance studies. To that end, one of the intergenic regions of VSV is replaced with the M^pro^ sequence. As both IGRs and M^pro^ produce individual gene products or proteins, their functions are interchangeable. To facilitate readouts, a monomeric form of GFP such as mWasabi can be added as an additional gene or into one of the virus genes as a marker. A monomeric fluorescent protein is preferable when choosing an intramolecular insertion site since it will not interfere with the function of the VSV gene (Fig. 1C).

**Figure 2:**
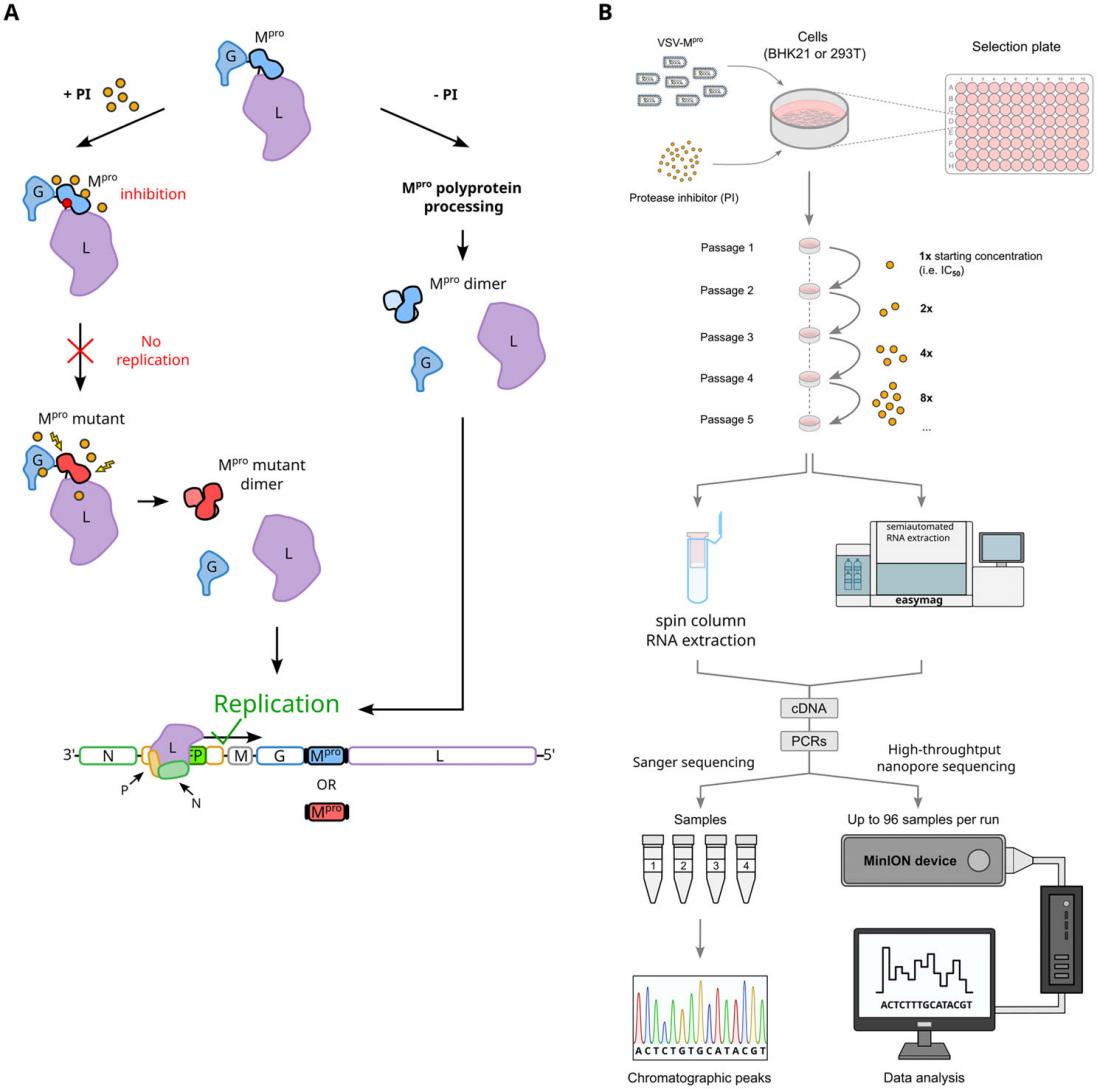
VSV‐M^pro^ resistance development and its assessment through sequencing. (**A**) VSV‐M^pro^ translates an artificial polyprotein, G:M^pro^:L. Without a specific M^pro^ inhibitor, the polyprotein is processed, and the virus can replicate. Adding a specific M^pro^ inhibitor arrests G:M^pro^:L processing, thus, VSV‐M^pro^ cannot replicate. Following acquisition of mutations that lead to resistance against the M^pro^ inhibitor, G:M^pro^:L is processed and VSV‐M^pro^ replicates. (**B**) Passaging VSV‐M^pro^ in the presence of increasing concentrations of a specific M^pro^ inhibitor leads to the selection of resistant mutants. Viral RNA is extracted from supernatants, reverse transcribed into cDNA, PCR‐amplified, and sequenced by Sanger sequencing or a suitable high‐throughput sequencing technique such as that provided by Oxford Nanopore Technologies.

The chimeric VSV‐M^pro^ translates an artificial polyprotein G:M^pro^:L. In the absence of a specific M^pro^ inhibitor, this polyprotein is processed into functional G, M^pro^ and L, facilitating virus marker gene expression and replication. Adding a specific M^pro^ inhibitor blocks polyprotein processing. However, the error‐prone L polymerase can serendipitously introduce a resistance mutation into M^pro^, thereby restoring polyprotein processing and subsequently replication (Fig. 2A). By repeated passaging in the presence of a specific M^pro^ inhibitor, e.g., in a high‐throughput, 96‐well format, mutant variants accumulate over time. Increasing the inhibitor concentration at every passage accelerates the selection process. Viral RNA is extracted from 96‐well supernatants, e.g., by spin filter columns or automated machines such as easyMAG. Viral RNA is then reverse transcribed into cDNA and the region of interest (M^pro^) is PCR‐amplified and sequenced. Among other sequencing techniques, Oxford Nanopore is practical for high‐throughput sequencing (Fig. 2B).

## USING VSV AS A PLATFORM FOR DIRECTED PROTEASE EVOLUTION

Vesicular stomatitis virus (VSV) has an error‐prone polymerase that on average mis‐incorporates 1 in 10,000 nucleotides. We can leverage that high error rate for directed evolution of a transgene incorporated into the VSV genome by imposing a selection pressure against that transgene. Examples of such transgenes are the main proteases (M^pro^s) of different coronaviruses. By replacing the intergenic region with a protease transgene (e.g., a coronavirus M^pro^), the protease's function becomes essential for VSV replication. Continuous passaging in the presence of an M^pro^ inhibitor selects for inhibitor‐resistant mutants, which can then be sequenced, tested, and structurally/mechanistically described.

VSV‐M^pro^ is a chimeric VSV variant where one of the intergenic regions was replaced by the main protease of SARS‐CoV‐2 (see Supporting Information, Supplemental sequence 1). Other coronavirus proteases can be used in this configuration as well, as previously shown with the M^pro^ of Middle East respiratory syndrome–related coronavirus (MERS‐CoV) (see Supporting Information, Supplemental sequence 2) (Krismer et al., 2024). Optionally, VSV‐M^pro^ can have a fluorescent protein in its genome, which facilitates viral readouts (see Supporting Information, Supplemental sequence 3).

### Materials

BHK21 (ATCC, cat. no. CCL‐10) or equivalent cells
HEK 293T (293tsA1609neo, ATCC, cat. no. CRL‐3216) or equivalent cells
Trypsin (Gibco, cat. no. 12604‐021, or equivalent)
Dulbecco's and Glasgow's modified Eagle medium (DMEM; GMEM), supplemented (see recipes)
FluoroBrite Dulbecco's modified Eagle medium (DMEM), supplemented (see recipe)
VSV‐M^pro^ (or other transgene of interest)
Low melting plaque agarose (see recipe)
Phosphate‐buffered saline (PBS) (Gibco, cat. no. 10010023, or equivalent)

T25, T75, T175 flasks, 10‐ to 15‐cm dishes, or equivalent vessels
LUNA II cell counter, Neubauer counting chamber, or equivalent device
6‐, 12‐, and 96‐well tissue culture plates
Media pools/containers/solution basin (BioRad, cat. no. ML10542, or equivalent)
Mono‐, 8‐ and/or 12‐channel pipettes and tips, 5‐ to 100‐µl, 30‐ to 300‐µl, and 100‐ to 1000 µl, or equivalent
Light microscope
Fluorescence microscope
EliSpot/FluoroSpot (C.T.L.) counter or equivalent measurement system (for fluorescence)
500‐µl, 1.1‐, and 2.2‐ml deep‐well plates for dilutions
Incubator, 5% CO_2_, 37°C
Microwave
Water bath
Vacuum pump
1.5‐ and 2‐ml microcentrifuge tubes
Supplemental files 1 and 2 (see Supporting Information)
GraphPad Prism software

### Day 0: Seeding of 293T or BHK‐21 cells

1 Grow HEK 293T or BHK‐21 cells in T25, T75, T175 flasks, 10‐ to 15‐cm dishes, or equivalent vessels.
2 Detach cells with trypsin, count and seed 5–10 × 10^3^ HEK 293T or BHK‐21 per well of a 96‐well plate on the day of infection or 1 day before.
  Cells can either be in suspension (same day seeding and infection) or adherent (seeding one day before infection). HEK 293T cells detach easily, therefore it is recommended to seed and infect on the same day. If cells should be evenly distributed for optimal counting (i.e., with an Elispot/Fluorospot counter), infection right after seeding is preferable. BHK‐21 cells are strongly adherent and will not detach when adding virus and inhibitors.
3 Seed cells in either 50 or 100 µl medium (supplemented DMEM for 293T cells; supplemented GMEM for BHK21 cells)  per well in 96‐well plates.
  If you do not have large amounts of your inhibitors, seeding a 50 µl volume can reduce the amount of inhibitor used. If cells are seeded in 50 µl, adjust the cell number accordingly, i.e., calculate twice the amount than for 100 µl. Example: 1 × 10^4^ cells per well in a 96‐well plate for 100 µl seeding volume = 1 × 10^5^ cells per ml; for 50 µl seeding volume = 2 × 10^5^ cells per ml.

### Day 0 to Day 1: Infection of cells (passage one)

4 Prepare virus at a multiplicity of infection (MOI) of 0.01 in a vessel with the appropriate size for the needed volume.
  The amount of virus stock required for this MOI can be calculated using the Excel sheet in Supplemental File 1 (see Supporting Information).
5 Add the calculated amount of inhibitor to the virus solution from the stock solution to achieve the desired concentration.
  Note that in the well, the inhibitor will be diluted 1:1, i.e., when 200 µM in 50 µl solution is mixed with 50 µl of cell/medium volume, the final concentration will be 100 µM. The MOI does not change with this 1:1 dilution, since MOI is not a concentration, but the absolute amount of virus adjusted to the absolute number of cells. Example: an MOI of 0.01 for 1 × 10^4^ cells are 1 × 10^4^ × 0.01 = 100 viral particles.
6 Pour virus/inhibitor mix into a pipetting pool and distribute the medium with either an 8‐ or 12‐channel pipette.
7 If cells were seeded just prior to infection, slap 96‐well plates from each side of the plates to homogeneously distribute cells. Check whether cells are now homogeneously distributed using an optical microscope.
  When this is the case cells will look homogeneously distributed and be mostly suspended in the supernatant. Typically, when cells are not evenly distributed, cell clusters are visible. For the first passage, cells, virus and inhibitor can also be mixed in one vessel before being distributed to 96‐well plates. In this case, adjust the inhibitor concentration to the desired final concentration (not twice the concentration as described above in step 5).

### Day 1 to Day 3: Assessing cell death and/or green fluorescence

Beginning with the day after infection, monitor cell death and/or fluorescence in each well daily. If a high‐throughput fluorescence microscope such as a CTL FluoroSpot counter is available, it can be used to screen for positive wells and quantify virus‐infected cells. A good rule of thumb is to have between 30% and 70% of wells that are positive. If all wells are positive, the inhibitor concentration might be too low to mount an effective selection pressure. High inhibitor concentrations might be cytotoxic, potentially masking the cytopathic effect (CPE) of the virus or killing the cells before viral infection can occur. Therefore, consider lowering the virus MOI and inhibitor concentration in case the inhibitor concentration required to achieve between 30% and 70% of positive wells is cytotoxic. If the cytotoxicity of the inhibitor is not yet known, it is recommended to first perform a cell viability assay (e.g., MTT assay).

### Day 2 to Day 4: Transfer virus for serial passaging

8 Prepare fresh 96‐well plates by seeding cells either a day before transfer or at the day of transfer in 50 µl supplemented GMEM (BHK‐21) or DMEM (HEK 293T).
9 If cells were seeded the day before the virus transfer, each 96‐well plate well contains 50 µl of medium. To fill up to the optimal volume of 100 µl and set the desired inhibitor concentration, add 50 µl of inhibitor solution at twice the final concentration of inhibitor. If cells are seeded on the day of the virus transfer, there are different options to set the desired inhibitor concentration.
  For example: (1) add inhibitor at the desired concentration to the cell suspension and seed 100 µl of the cell suspension, or (2) seed cells in 50 µl to then add 50 µl of inhibitor solution with twice the desired concentration of inhibitor.
10 Transfer a small volume of supernatant from the previous passage plate (where viral progeny is present), e.g., 1, 2 or 5 µl, to a new plate with fresh cells.
  An estimate of the amount of virus in a well can be made by either looking at the cytopathic effect or GFP expression (in the case of a virus with such a reporter gene). A small volume is advised to avoid high MOIs and resulting defective interfering viral particles. To ensure low enough MOIs without actually titrating every passage, a prior replication kinetic and stock titration can help to estimate the rough number of viral particles arising at every passage. VSV replicates to very high titers of 10^10^/ml, but chimeric variants might be attenuated and therefore replicate to lower titers. The transfer volume can be adjusted based on this information, e.g., to‐be‐transferred wells can be diluted in a 96‐well plate before the passage.
11 To select and enrich mutants, repeat steps 1 to 3 at least five times with increasing inhibitor concentrations.
  The increment can be chosen according to how many wells are virus positive per passage. If almost all wells are negative, the increment was too high. If almost all wells are positive, the increment could be higher for a more effective selection pressure. A typical starting concentration can be the IC_50_ of the transgenic VSV with the inhibitor of choice. A typical increment can be doubling the inhibitor concentration at every passage.
12 If the concentration of inhibitor becomes too high (and therefore cytotoxic), it is recommended to increase the concentration in smaller steps (i.e., start, 30 µM; serial passaging, 50, 70, and 90 µM; final passage, 100 µM).

### Plaque‐purification of mutant viruses

After selecting and enriching mutants, purify RNA and proceed with Nanopore sequencing as described in Support Protocol 1. Mutants of interest can be plaque‐purified for subsequent resistance testing.

**Figure 3:**
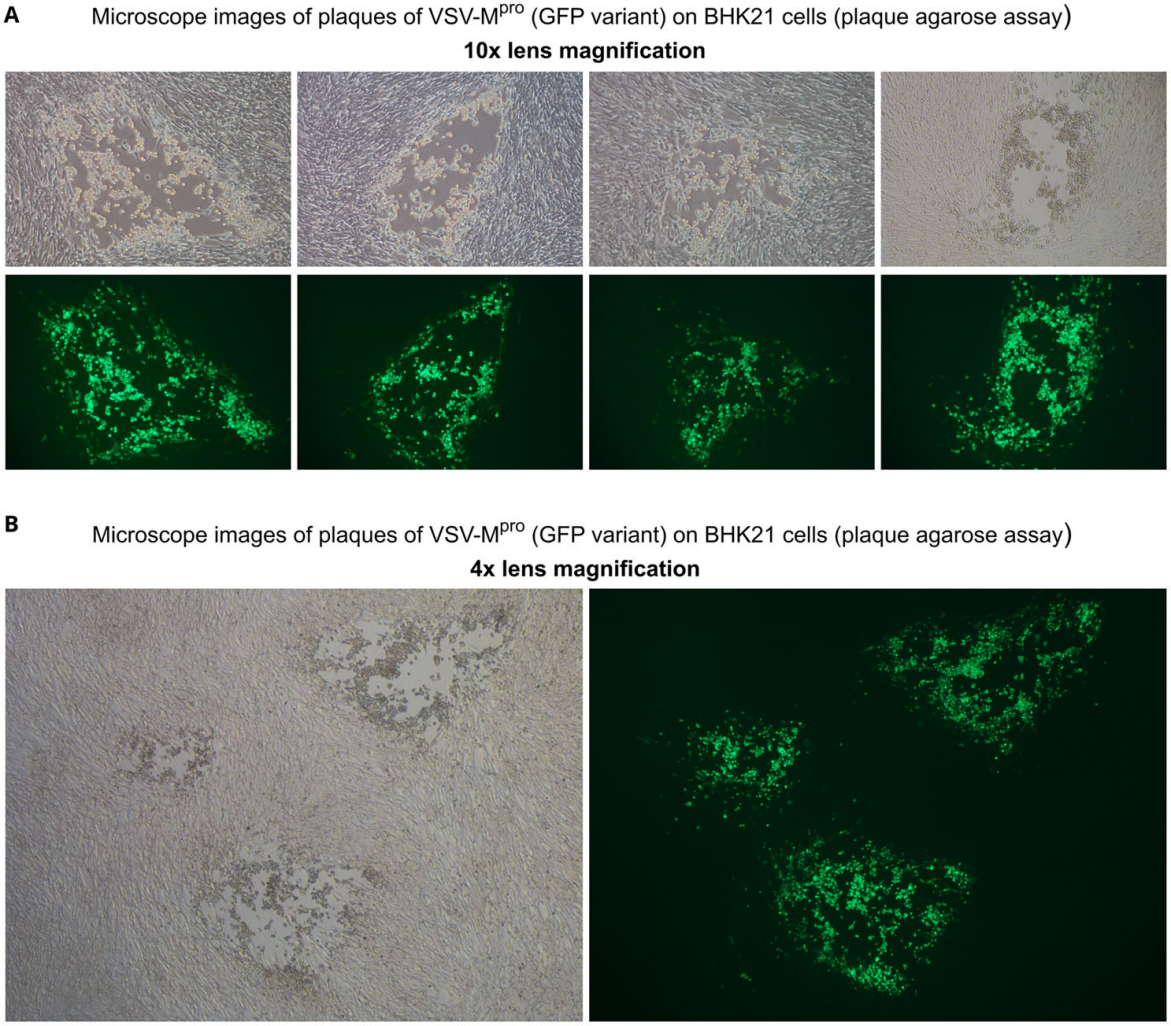
Plaque‐purification of mutant viruses. (**A**) After 1‐3 days, plaques appear in the cell layer. Plaques are holes in the cell layer containing rounded‐up/dead cells. They can be distinguished from other holes, e.g., unevenly seeded cells, scratch or pipetting marks, because no new cells will grow into the plaque. A virus expressing a fluorescent protein further facilitates plaque‐picking. (**B**) Isolation of virus from large plaques can help to pick viruses with better kinetic properties and facilitate picking in general. Observation using a lower magnification (40× magnification) allows an overview of different plaque sizes on the plate.

13 Seed BHK‐21 cells in 6‐ or 12‐well plates one day before infection.
  2 × 10^5^ cells per 6‐well and 1 × 10^5^ per 12‐well yield a sparce cell layer. Too‐dense cell layers will decrease plaque sizes, which makes plaque picking more difficult, albeit not impossible (see Supporting Information, Fig. S1).
14 At the day of infection, prepare serial dilutions of the desired well using deep‐well plates. Starting with 1:100 and going up to 1:1 × 10^7^ is a typical range.
15 Infect cells with 1 ml of each serial dilution step per well when using a 6‐ or a 12‐well plate. Using smaller plates and less volume is possible when adjusting the formula to the new volume.
16 Incubate for 1 hr at 37°C.
17 In the meantime, heat low‐melting plaque agarose [1.8% (w/v) in PBS] in a microwave until it is fully dissolved.
18 Cool low‐melting plaque agarose in a water bath to 37°C. Mix low‐melting agarose 1:1 with pre‐warmed medium (supplemented DMEM for 293T cells; supplemented GMEM for BHK21 cells). Optionally, the medium can contain protease inhibitor to suppress wild type virus–containing plaques.
19 Remove medium from 6‐ or 12‐well plates with a vacuum pump.
20 Add medium/plaque‐agarose mix.
21 Let the plaque agarose solidify at room temperature.
22 Once solid, the plaque agarose will not become liquid again in a 37°C incubator, even though it can be maintained as a liquid at 37°C.
23 Incubate at 37°C, 5% CO_2_ for 1 to 2 days or until plaques become visible (Fig. 3A‐B).

24 Seed cells in 6‐ or 12‐well plates again for inoculation and virus culture on the day before the plaque picking.
25 Pick plaques by piercing through the agarose layer with a pipette tip until it touches the plastic. Move the tip slightly in small circles.
26 Inoculate each well of the previously seeded 6‐ or 12‐well plate with one plaque by dipping the pipette tip into the medium and pipetting up and down.
27 Grow virus several days until most cells are dead and/or fluorescent.
28 Collect virus in 1.5‐ or 2‐ml Eppendorf tubes and freeze them at –80°C.

### Virus titration with TCID_50_

**Figure 4:**
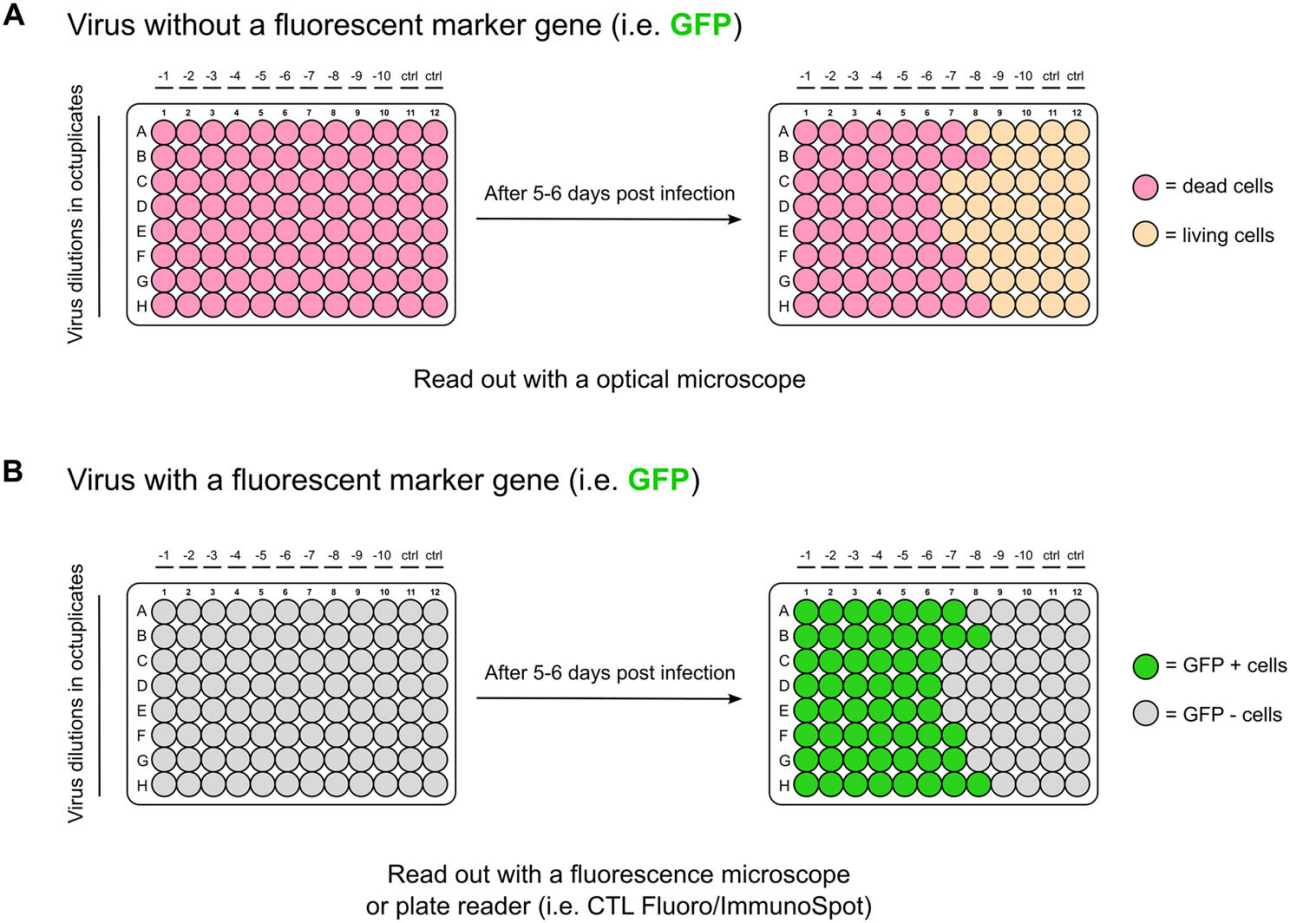
Virus titration with TCID_50_. (**A**) Titration of a VSV variant without fluorescent protein. Cells are seeded at very low density. Wells where VSV quickly kills the few present cells will not grow dense enough to exhaust the medium. Phenol red therefore remains pink. Wells where VSV kills cells later due to low inoculation particle number will grow dense and turn phenol red to yellow. The color difference helps to concentrate on relevant wells to read out viral titers. However, for exact titers, a microscopic inspection on the border of pink and yellow wells is still necessary. (**B**) A fluorescent protein supports the titer readout.

29 Seed 1 × 10^3^ BHK‐21 cells in 100 µl per well in 96‐well plates one day before infection.
30 Prepare serial dilutions of the virus in either logarithmic (1:10^1^ = 1:10) or half‐logarithmic (1:10^0.5^ = 1:3.162) dilution steps in a deep‐well plate. For example, logarithmic dilutions can be done with 900 + 100 µl and half‐logarithmic dilutions with 900 + 416 µl.
31 Transfer the volume from the deep well plate to eight 96‐well plates with an 8‐ or 12‐channel pipette. With an 8‐channel pipette, start from the highest dilution, i.e., the lowest amount of virus on the right side of the plate to be able to reuse the tips.
32 Incubate for up to 6 days at 37°C, 5% CO_2_.
33 Print template sheet provided in Supplemental file 2 (see Supporting Information) and read out cell death.
  We recommend the use of medium containing phenol red to expedite well counting. The cells in wells containing virus will be killed, therefore the medium will remain red. Wells containing live cells, and therefore no virus, will turn yellow due to a pH change of the medium (Fig. 4A). If GFP is additionally expressed by the virus, the titer can be determined by reading the fluorescence using an Fluoro/ImmunoSpot counter, or equivalent (Fig. 4B).

34 Fill out dead and alive well count in Supplemental file 2 (see Supporting Information) to retrieve viral titers.

### Dose response assay with FluoroSpot readout

**Figure 5:**
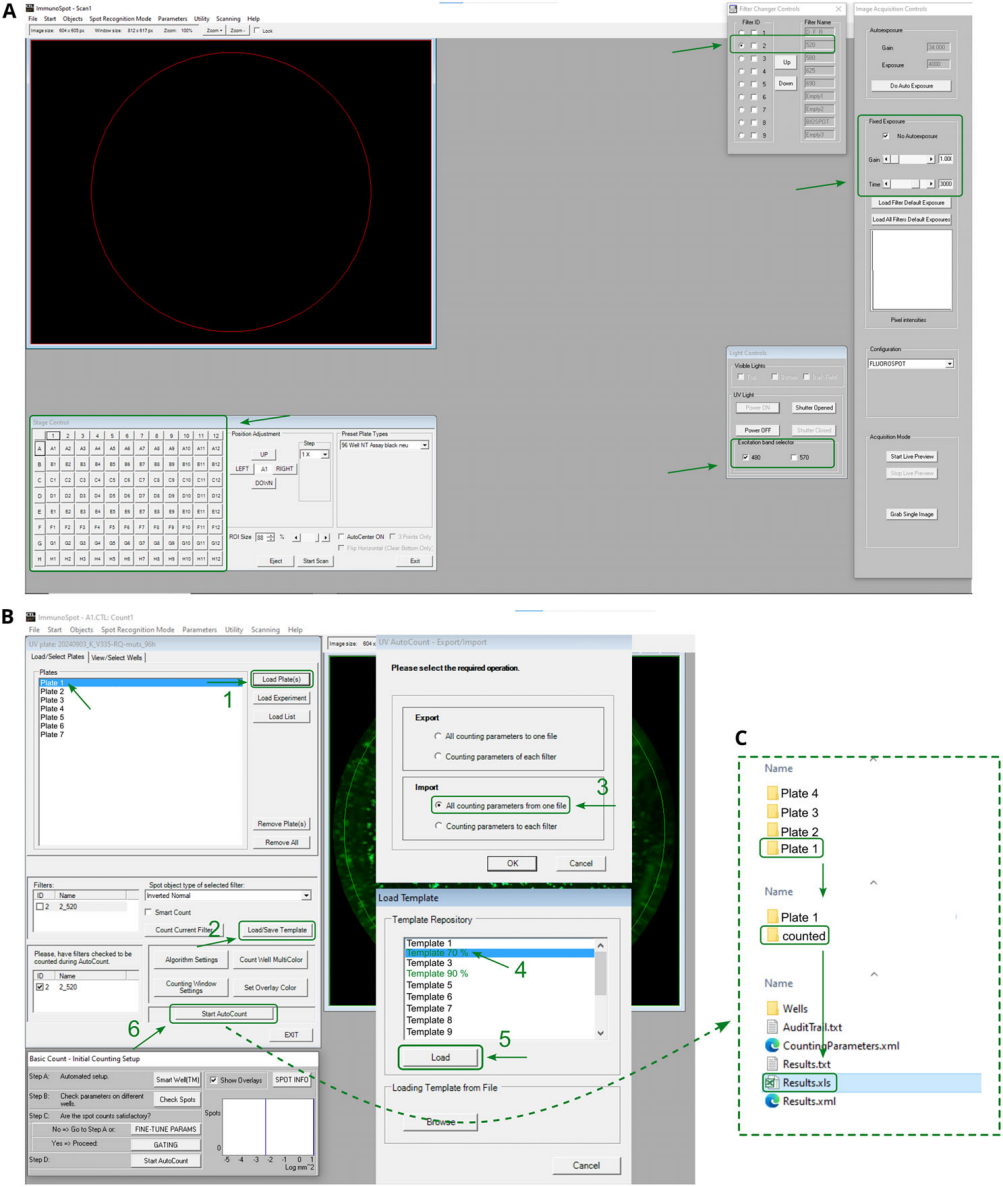
CTL FluoroSpot X Suite for plate scanning and spot counting. (**A**) Scan suite. Stage control: 96‐well control panel. (1) Eject instrument's plate tray and place the plate to be analyzed, and immediately press “Load” button, which appears at the same position as the “Eject” button. (2) Filter change controls: filter ID 2 for green fluorescence. (3) Image acquisition controls: no Autoexposure; gain = 1.00; time = 3000. Both gain and time are chosen depending on the strength of the fluorescent signal. (4) Start scan. Choose your destination folder and the name of the new folder. (**B**) Counting suite. (1) Load plate(s): select the plate from the previous scan. (2) Load/save template. (3) Import all counting parameters from one file. (4) Select the template. (5) Load. (6) Start AutoCount. (**C**) Find results in folder under the name given by the experimenter.

35 After 24 to 72 hr, check fluorescence signal with a fluorescence microscope.
36 Spots can be counted in a Fluoro/ImmunoSpot or equivalent device.
37 When using a Fluoro/ImmunoSpot with the manufacturer‐provided software CTL switchboard, scan 90% (if medium is removed) or 70% (if medium is not removed) of each well area concentrically to exclude reflection from the well edges and normalize to the full area. Apply automatic fiber exclusion while scanning. Medium can be either supplemented DMEM or GMEM.
38 The excitation wavelength for mWasabi/GFP is 488 nm, use the 520 nm filter to measure fluorescence (Fig. 5A). Count spots with counting templates. The counting parameters of these templates can be defined once, saved, and then imported before the run (Fig. 5B).

39 Alternatively, spot counts can be performed with an equivalent counter such as BZ‐X810 All‐in‐One fluorescence microscope from Keyence, a Cytation|1 Imaging reader from BioTek, or any equivalent device.
40 When using a Fluoro/ImmunoSpot counter, the software requests a name for the experiment before the scan begins. Scanned images are stored in a folder on the computer supplied with the reader. The software creates a folder that has the same name as the user has given the experiment.
41 Results are put out in Excel after counting and placed into the same folder.
42 Make use of the software‐integrated quality control steps like manual fiber removal after counting.
43 After quality control, another folder is created for results with quality control edits displayed in red.

### Data analysis

44 CTL Fluoro/ImmunoSpot software provides Excel sheets containing data. Depending on the reader's software, different export options might be available.
45 Data can be analyzed using GraphPad Prism to calculate IC_50_ values.
46 When using GraphPad Prism, choose XY table format and go to “Data Tables.”
47 Input used inhibitor concentrations, sample names and spot counts.
48 Press “analyze” and choose “Nonlinear regression (curve fit).”
49 On the right, choose the samples to be analyzed.
50 One option is to use the following equation (sigmoidal, 4PL):
$$Y=B+\frac{T-B}{1+{\left(\frac{IC_{50}}{X}\right)}^{HillSlope}}$$
  Where T is the top of the curve (plateau), B is the bottom of the curve, X is the concentration, and HillSlope is the steepness of the curve when the signal is decreasing.
51 Go to results to retrieve IC_50_ values.
52 Go to graphs to retrieve curve fittings.

## DOSE RESPONSE ASSAY WITH TCID_50_ READOUT

If the VSV variant used for directed evolution does not contain a fluorescent protein gene, TCID_50_ can be used to read out effects on viral titers achieved by inhibitor‐treated mutants.

### Materials

See Basic Protocol

1 Follow Basic Protocol, steps 29 to 34, to titrate wild‐type vs mutant VSV‐M^pro^ variant supernatants after a dose response experiment.

## Support Protocol 1 A PIPELINE FOR HIGH‐THROUGHPUT VSV SEQUENCING

The easy handling of VSV as mutation tool allows for the generation of large pools of mutants in short periods. Therefore, setting up a high‐throughput sequencing pipeline is sensible for mutation studies as described in this protocol. The pipeline we established for this protocol comprises RNA isolation, cDNA synthesis, PCR on the region of interest, and Nanopore sequencing. In our experience, RNA purification and sequencing were bottlenecks, whereas cDNA synthesis and PCR were simple to scale. RNA purification can be done with RNA binding columns such as provided by E.Z.N.A. viral RNA kit (Omega Bio‐Tek). However, purification of hundreds of samples by hand is impractical. We therefore adapted a semiautomated RNA extraction strategy with an easyMAG instrument (BioMerieux). Then, cDNA is synthesized from isolated viral RNA by RevertAid RT reverse transcription kit (Thermo Fisher Scientific). For PCR amplification of regions of interest, we used the high‐fidelity polymerase Q5 (New England Biolabs). However, any equivalent polymerase with a low error rate is suitable at this step. Sanger sequencing is possible for smaller genes of interest, e.g., <1000 bp. The size limitation of Sanger sequencing becomes impractical above that, since the same sample must be sequenced with multiple primers. We therefore established a Nanopore (Oxford Nanopore Technologies) sequencing workflow, which is size‐independent and allows up to 96 samples per run and sequencer.

### Materials

VSV plates (see Basic Protocol)
E.Z.N.A. viral RNA kit (Omega Bio‐Tek Inc, cat. no. R6874, or equivalent)
NUCLISENS easyMAG high‐throughput nucleic acid extraction device
RevertAid cDNA synthesis kit (Thermo Fisher Scientific, cat. no. K1622, or equivalent)
Q5 polymerase (New England Biolabs, cat. no. M0491S)
H_2_O, sterile, molecular biology grade
Rapid barcoding kit (Oxford Nanopore, cat. no. SQK‐RBK110.96, or later versions)

1.5‐ml microcentrifuge tubes
0.2‐ml thin‐walled PCR tubes
Thermal cycler or heat block
Spectrophotometer (e.g., Nanodrop)
MinION Mk1B sequencer (Oxford Nanopore)
MinKNOW software
Geneious Prime (or equivalent clone manager software)

### RNA purification with E.Z.N.A. viral RNA kit or semi‐automated RNA purification of VSV‐M^pro^ genome

The E.Z.N.A. viral RNA kit is a standard RNA purification kit with the extra step of adding carrier RNA. Carrier RNA can improve the yield of low‐abundance viral RNA by acting as a co‐precipitant. It can also act as a decoy for RNases. Since VSV produces large amounts of RNA and is extremely cytolytic, the RNA quantity and quality is usually not a concern. Therefore, other equivalent RNA purification kits are also applicable at this step. For the protocol steps, consult the manual provided with the kit and available online. As an alternative method, a semi‐automated system such as easyMAG expedites high‐throughput RNA production.

#### When using manual viral RNA isolation kits

1a Supernatants from each well of the plates where VSV was passaged (VSV plates) are collected and transferred into 1.5‐ml microcentrifuge tubes and stored until further processing.
  For short storage times (1 to 2 days), supernatants can be kept in the fridge; for longer storage times, samples should be stored at least at –20°C, but ideally at –80°C.
2a RNA isolation is carried out as outlined in the kit's protocol.
3a After elution (typically in 50 µl of RNase free water), RNA samples are stored at –80°C.

#### When using semi‐automated RNA isolation method

1b Supernatants from each well are divided into PCR tubes or 96‐well round bottom plates up to 200 µl volume for easy/high‐throughput pipetting of large numbers of samples.
2b RNA isolation is carried out following the device manufacturer's instructions.
3b After elution (typically in 50 to 60 µl of manufacturer's proprietary elution buffer), RNA samples are stored at –80°C.

### cDNA synthesis with RevertAid RT

4 The manufacturer's instructions for RevertAid are to use ≤5 µg of RNA. If the RNA yield is low after the purification or the RNA degraded, use the highest amount of RNA possible, i.e., do not add extra water, but only sample to the mix.
5 The manufacturer recommends 20 µl reaction volume (Table 1). However, to scale up and save reagent, 10 µl are also possible (in this case, all the reagent volumes will be divided by 2).

**Table 1:** cDNA Synthesis with RevertAid RT, Reaction Mix and Cycler Program

Reaction mix	Per reaction (µl)
RT reaction buffer	4
RiboLock RNase inhibitor (20 U/µl)	1
10 mM dNTP mix	2
Random hexamer primer	1
RevertAid M‐MuLV RT (200 U/µl)	1
RNA	11
Total volume	20

Cycler program time	Cycler program temperature
5 min	25°C
60 min	42°C
5 min	70°C
Hold	4°C

### PCR with Q5 polymerase

6 PCRs on the region of interest are best performed with a high‐fidelity enzyme to avoid the introduction of false positive mutations. For example, use Q5 or an equivalent enzyme with the mix (see Table 2) and thermo‐cycler program.

**Table 2:** PCR with Q5 Polymerase, Reaction Mix

Reaction mix	Per 25 µl reaction (µl)	Per 50 µl reaction (µl)
H_2_O	15.75	31.5
5× Q5 reaction buffer	5	10
DMSO	0.5	1
10 mM dNTP mix	0.5	1
Primer forward	1	2
Primer reverse	1	2
Q5	0.25	0.5
cDNA	1	2
Total volume	25	50

7 The manufacturer recommends either 25 or 50 µl reaction volumes (Table 2). To scale up to many samples and save reagent, reduce the volume to ≤25 µl.
8 To set up the PCR program (Table 3), make use of calculation tools to find the optimal annealing temperature based on the primer pair, e.g., https://tmcalculator.neb.com/#!/mainonline.

**Table 3:** PCR with Q5 Polymerase, Cycler Program

Step	Time	Temperature
Initial denaturation	30 s	98°C
40 cycles of:		
Denaturation	5 s	98°C
Annealing	20 s	Dependent on primers
Elongation	30 s/kb	72°C
Final extension	5 min	72°C
Hold	∞	4°C

9 The manufacturer recommends 20 to 30 s/kb for elongation. It is also possible to increase it to 1 min/kb.

### Sequencing with Nanopore

Completed PCR reactions will return an amplicon that comprises the M^pro^ sequence embedded in the transgenic VSV, e.g., VSV‐M^pro^ genome containing SARS‐CoV‐2 Wuhan‐1 M^pro^ (see Supporting Information, Supplemental sequence 1). VSV versions with main proteases from other viruses can also be amplified, such as VSV‐MERS‐M^pro^ containing the main protease of MERS‐CoV (see Supporting Information, Supplemental sequence 2). To facilitate the readout, transgenic VSV can further contain a fluorescent marker gene as intramolecular insertion in the phosphoprotein gene, such as VSV‐PmNeon‐M^pro^ (see Supporting Information, Supplemental sequence 3). Depending on the originating virus, the amplicons will have a different length (in base pairs), forward and reverse primers and a reference sequence. Therefore, the sequencing analysis (below) must be adapted accounting for these differences.

10 Prior to starting the sequencing, the DNA concentration of the PCR samples must be determined using a spectrophotometer (e.g., Nanodrop).
11 DNA dilutions should be prepared as follows:
  - X µl of each PCR sample are calculated to reach a final concentration of 200 ng/µl.
  - Then (18 – X) µl of water (molecular biology grade) are transferred to a clean PCR tube.
  - Finally, X µl of PCR sample are diluted in water to reach the desired concentration.
12 Prepare the sequencing library using the Rapid barcoding kit.
  The Rapid barcoding kit allows for high‐throughput processing and multiplexing of up to 96 samples per sequencing run.
13 The prepared library is loaded on a MinION R9.4.1 flowcell, following manufacturer's instructions.
14 The sequencing is performed on a MinION Mk1B sequencer device with standard settings. Q score threshold is set to 8.
15 The sequencing run can be live monitored through the MinKNOW software and the experiment can be stopped at any time. Usually, sequencing is stopped when the number of reads for each barcode is sufficiently high (i.e., 10,000 reads).
16 Raw data is stored as .pod5 files (in the form of electrical signals), basecalled (the electrical signals are translated into nucleotides) using the super accuracy model (Guppy version 6.5.7, Oxford Nanopore). At the same time, sequences are demultiplexed (divided according to the barcodes, from 1 to 96) and the barcodes and the adapter sequences are trimmed.
17 Fastq files are filtered to a PHRED quality score of ≥Q15 and length, which is set depending on the amplicon length, using SeqKit version 2.4.0 (Shen et al., 2016). Minimum = 200 bp to a maximum = amplicon length.
18 Multiple .fastq files belonging to the same barcode are merged into one single .fastq file for each barcode, before the analysis pipeline is started.
19 Filtered sequences are aligned to the appropriate reference sequence using minimap2 (version 2.22) (Li, 2018). The reference sequence must be indexed beforehand with the terminal command <<samtools faidx reference_sequence.fasta>>.
20 Sequences are sorted and indexed using SAMtools version 1.13 (Danecek et al., 2021).
21 SAMtools is also used to check sufficient depth (>300 reads).
22 To find single nucleotide variations, LoFreq (v2.1.3.1) (Wilm et al., 2012) was employed for variant calling.
23 The resulting .vcf files are imported to Geneious Prime 2023.0.1 and called variants are manually checked for plausibility.

## Support Protocol 2 RESCUE OF VSV

The generation of a new virus from plasmid DNA is typically referred to as “rescue.” As a negative sense RNA virus, the rescue of recombinant VSV is a non‐trivial protocol that is more challenging than that of positive sense RNA viruses, as negative‐strand RNA cannot be infectious per se, other than positive‐strand RNA (Wang et al., 2024). The difficulty is routed in the negative sense RNA genome structure, which is not directly recognized at the ribosome to produce viral proteins. Instead, virus particles must import certain proteins, such as the polymerase, called “large protein” or “L” in VSV. Historically, to overcome the issue of the negative sense genome, a helper‐virus was used, which had to be removed after VSV was generated. More recently, helper‐virus‐free protocols were developed that instead rely on helper plasmids (Witko et al., 2006). In this protocol, a T7 polymerase (see Supporting Information, Supplemental sequence 4), VSV‐N (see Supporting Information, Supplemental sequence 5), ‐P (see Supporting Information, Supplemental sequence 6), ‐L (see Supporting Information, Supplemental sequence 7) and optionally ‐M and ‐G are transfected into cells together with a plasmid containing the entire genome of the VSV variant of interest. The helper plasmids T7, N, P, L and optionally M, G are recognized by cellular DNA‐dependent RNA polymerases, which synthesize mRNAs. After its translation, T7 transcribes an unmodified (no cap or poly‐A) RNA from the full‐genome VSV plasmid. Additionally, ribozymes encoded at the start and end of the VSV genome plasmid can improve the rescue efficiency by cleaving the transcribed RNA at defined sequences to yield a precise VSV antigenome (plus strand RNA version of the genome). This antigenome is recognized by the L‐P transcription and the L‐P‐N replication complexes to transcribe viral mRNAs and genomic RNA, respectively.

### Materials

HEK 293T (293tsA1609neo, ATCC, CRL‐3216) or equivalent cells
DMEM, supplemented (see recipe)
VSV rescue helper plasmids T7, N, P, L (see Supporting Information, Supplemental sequences 4 to 7)
Transgenic plasmids of VSV (e.g., Supplemental sequences 1 to 3, see Supporting Information)
H_2_O, sterile, molecular biology grade
2.5 M CaCl_2_ (see recipe)
2× HEPES‐buffered saline (HBS) (see recipe)
25 mM chloroquine (see recipe)
Low melting plaque agarose (see recipe)

10‐cm tissue culture dishes
Monopipettes and tips, 5‐ to 100‐µl, 30‐ to 300‐µl, and 100‐ to 1000‐µl, or equivalent
1.5‐, 5‐, 15‐, and 50‐ml sterile reaction tubes
Vortexer
Cell culture incubator
Water bath
Vacuum pump
5‐, 10‐, and 25‐ml sterile pipettes
6‐, 12‐, and 96‐well tissue culture plates

### Day 1: Seeding of HEK 293T cells

1 Seed 2–3 × 10^6^ HEK 293T cells in 10‐cm dishes in 8 ml supplemented DMEM.
2 Check cell density the next day. The cells should be ∼50% to ∼70% confluent.

### Day 2, evening: Transfection via Ca_2_PO_4_ /rescue

3 Prepare helper and genome plasmid mix (Table 4, 1 sample).
  A VSV rescue can be done with any transfection method. However, in our hands, Ca_2_PO_4_ produced the most reliable results. The more cells are transfected, the higher the chance of the rescue succeeding. Therefore, scaling up to 10‐cm dishes using the DNA quantities from Table 4 increases the chances of a successful rescue. Large‐scale Ca_2_PO_4_ transfections are typically cheaper than lipid‐based methods, as the components can be made in‐house (see recipe).

**Table 4:** Transfection via Ca_2_PO_4/_Rescue Plasmid DNA Amounts

Component	Amount (µg)
T7 polymerase	10
VSV‐N	2.4
VSV‐P	1.8
VSV‐M (optional)	1
VSV‐G (optional)	1
VSV‐L	0.7
VSV full genome plasmid	10

4 Pipet the following into a 1.5‐ml reaction tube (500 µl final volume) and mix by vortexing [for one 10‐cm dish (59 cm^2^)]:
  Prepared plasmid DNA mix (Table 4)
  Sterile water to bring the volume up to 450 µl
  50 µl 2.5 M CaCl_2_.
5 Pipet 500 µl of 2× HBS into a 50 ml reaction tube.
6 Place the 50 ml reaction tube on a vortex mixer.
7 Using a 1000 µl pipette, add the DNA/CaCl_2_ mixture slowly and dropwise under vigorous vortexing.
8 Vortex for 15 to 20 s.
9 Incubate the mixture at room temperature for 20 min to allow for the formation of calcium‐phosphate‐DNA precipitates.
10 During the incubation time, mix 2 ml supplemented DMEM with 10 µl of 25 mM chloroquine stock solution. Carefully add the mix to the 10‐cm tissue culture dish with cells seeded the previous day and move the dish gently to mix (10 ml total volume in the dish; 25 µM chloroquine final concentration). If the 20 min incubation time is not yet over, put the cells back in the incubator.
11 Thoroughly vortex the DNA precipitation mix or mix by pipetting up and down a few times.
12 Tilt the 10‐cm dish and add the transfection mix drop‐wise to the accumulated medium on one side with a 1000 µl pipette. Take care not to detach cells while adding transfection mix.
13 Gently rock the culture vessel back‐and‐forth and from side‐to‐side to evenly distribute the calcium‐phosphate‐DNA complexes. Do not swirl or rotate the dish, as this may cause uneven distribution.
14 Culture cells in the incubator for 6 to 16 hr or overnight.

### Day 3, morning/after 6 to 16 hr: Chloroquine removal

15 Prewarm supplemented DMEM to 37°C in the water bath.
16 Carefully remove the medium from the dish while holding it at an angle using a vacuum pump or a serological pipette. Discard the medium.
17 Add 10 ml fresh medium (supplemented DMEM) without chloroquine. To prevent flushing away the cell layer, this is best done with the dish lying flat and the serological pipette, touching the outer rim on the dish.

### Days 4 to Day 10: Observation and co‐culture

18 When cytopathic effect or fluorescence occurs, seed new, untransfected cells in 6‐well plates or 10‐cm dishes.
19 Transfer ∼5 ml of supernatant from the rescue to the fresh cells and refill the rescue medium.
20 When the fresh cells are also showing cytopathic effect or fluorescence, proceed with plaque‐purification.

### Plaque purification and stock production

To purify a replication competent virus from the mix of genomes and defective interfering viral particles that form during rescues, perform a plaque purification. For this procedure, strongly adherent cells are preferable, as the process includes removing medium and adding plaque agarose, which can detach weakly adherent cells. If the virus encodes a fluorescent protein, a uniform cell layer is less critical since plaques will also be visible via fluorescence. Follow Basic Protocol, steps 13 to 28.

## REAGENTS AND SOLUTIONS

### CaCl_2_, 2.5 M

27.745 g CaCl_2_ (Roth, cat. no. HN04.3, or equivalent)
100 ml sterile H_2_O
Sterile filter with a 0.22‐µm filter (Merck, cat. no. SLGPR33RS, or equivalent)
Store up to 10 years at –20°C or 1 year at 4°C

### Chloroquine, 25 mM

79.97 mg chloroquine (VWR, cat. no. IC0219391950)
10 ml sterile H_2_O
Sterile filter with a 0.22‐µm filter (Merck, cat. no. SLGPR33RS, or equivalent)
Store up to 10 years at –20°C or 1 year at 4°C
  Chloroquine solution is used in Ca_2_PO_4_ transfections for virus production. To reconstitute Ca_2_PO_4_, 2× HBS and 2.5 M CaCl_2_ must be prepared.

### DMEM, supplemented

500 ml DMEM (Gibco, cat. no. 41965062, or equivalent)
50 ml fetal calf serum (FCS), heat‐inactivated (Gibco, lot no. 2563339H, or equivalent)
5 ml l‐glutamine or Glutamax (Gibco, cat. nos. 25030081, 35050028, or equivalent)
5 ml penicillin/streptomycin (Gibco, cat. no. 15140‐122, or equivalent)
5 ml non‐essential amino acids (Gibco, cat. no. 11140‐035, or equivalent)
5 ml sodium pyruvate (Gibco, cat. no. 11360‐070, or equivalent)
Store up to 3 months at 4°C
  HEK 293T cells can be grown in DMEM supplemented medium if FluoroSpot readout is not used, or if the medium will be removed before readout.

### Fluorobrite DMEM, supplemented (used to grow HEK 293T cells)

500 ml FluoroBrite (Gibco, cat. no. A1896701, or equivalent)
50 ml FCS, heat‐inactivated (Gibco, lot no. 2563339H, or equivalent)
5 ml l‐glutamine or Glutamax (Gibco, cat. nos. 25030081, 35050028, or equivalent)
5 ml penicillin/streptomycin (Gibco, cat. no. 15140‐122, or equivalent)
5 ml non‐essential amino acids (Gibco, cat. no. 11140‐035, or equivalent)
5 ml sodium pyruvate (Gibco, cat. no. 11360‐070, or equivalent)
Store up to 3 months at 4°C
  HEK 293T cells are grown in FluoroBrite DMEM supplemented medium whenever multiple FluoroSpot readouts (especially with fluorescent proteins that are disturbed by the phenol red) are intended.

### GMEM, supplemented

500 ml GMEM (Gibco, cat. no. 11710035, or equivalent)
50 ml FCS, heat‐inactivated (Gibco, lot no. 2563339H, or equivalent)
25 ml tryptose phosphate broth (Sigma, cat. no. T8159, or equivalent)
5 ml penicillin/streptomycin (Gibco, cat. no. 15140‐122, or equivalent)
Store up to 3 months at 4°C
  GMEM is used to grow BHK21 cells.

### HBS, 2×

Dissolve 4.77 g HEPES (Roth, cat. no. HN77.6, or equivalent) in 200 ml. Add 3.28 g NaCl (Roth, cat. no. P029.5, or equivalent). Add 4.25 mg Na_2_HPO_4_ (Roth, 4 cat. no. 984.1, or equivalent). Adjust to pH 7.1 with NaOH, sterile filter with a 0.22‐µm filter (Merck, cat. no. SLGPR33RS, or equivalent) and store up to 1 year at 4°C or 10 years at –20°C.

### Low melting plaque agarose

1.8 g low melting plaque agarose powder (Sigma, cat. no. A9414‐5G)
100 ml PBS (Gibco, cat. no. 10010023, or equivalent)
Heat in a microwave until a completely homogenous solution
Store up to 1 year at room temperature

## COMMENTARY

### Critical Parameters

#### Multiplicity of infection

The multiplicity of infection (MOI) is one of the critical parameters for successful mutant selection. In general, infecting at a low MOI is preferred in passaging a virus because high MOIs will lead to the accumulation of defective interfering genomes and particles (Bora et al., 2019). In the particular case of a mutant selection, the low MOI and also a low transfer volume has the additional purpose of increasing the selection pressure and purifying the mutant from the parental genome over time. Especially when working with an inhibitor that is not very potent, MOIs of 0.01 and lower will still mount an effective selection pressure.

#### Inhibitor toxicity

Newly designed inhibitors, especially if they have not yet been licensed as drugs, might still have toxicity issues that need to be addressed at some point in the drug's development. For the selection process, toxicity will introduce a ceiling above which the inhibitor cannot be applied to produce or increase a selection pressure. If toxicity is an issue, more robust cell lines can be used to perform selection experiments. The resistance of the cell line to the inhibitor is a particular parameter that might not be known before and must be determined empirically by the lab interested in using a particular inhibitor. In our experience, BHK21 cells are very robust, more so than HEK 293T cells. Another parameter to lower inhibitor concentrations while retaining a strong selection pressure is the previously described MOI.

#### RNA quality

To generate reliable sequencing results, RNA quality is an important factor. As RNA can be inherently unstable, quick processing and immediate freezing are good practices to avoid degradation. VSV in general produces very large quantities of RNA as it is a fast‐replicating virus (Reed, 2018). We found that the specialized viral RNA kit described in the protocol yields usable amounts of VSV RNA. The semi‐automated high‐throughput method further expedited RNA purification. After successful cDNA synthesis, the least problematic step is the PCR to amplify the region of interest, as it is very robust.

#### DNA in Nanopore sequencing and sequencing depth

The preparation of the DNA library should be done with ∼200 ng per sample. Besides the obvious issue of having not enough DNA and therefore insufficient sequencing depth to reach quality cut‐offs in the sequencing pipeline, overloading the DNA library preparation is also a serious issue. The barcoding transposases used in the protocol only have a certain capacity and limited incubation time (5 min). Therefore, exceeding DNA limits will result in non‐barcoded DNA entering the Nanopore channels, leading to unusable data. An uneven distribution of DNA quantity between samples can lead to occupancy of all pores by a dominant sample, hiding the other sequences.

### Troubleshooting

Table 5 describes possible problems that may arise from the protocols outlined above and proposed solutions.

**Table 5:** Troubleshooting for Using VSV as a Platform for Directed Evolution

Problem	Possible cause	Solution
Positive signal in all wells, no distinction between inhibitor negative/positive wells	MOI too high	At high MOIs, the virus will kill all cells and produce fluorescent signals; lower the MOI, re‐titrate the virus, or ensure MOI was calculated correctly
No mutants after several rounds of selection	MOI, too high, inhibitor concentration too low	If the inhibitor chosen for the selection experiments is not potent enough to achieve an adequate selection pressure at the highest possible concentration that is not yet cytotoxic, try lowering the MOI to 0.001
Excessive or no precipitate after Ca_2_PO_4_ transfection	Incorrect pH of 2× HBS	Measure buffer concentration again and make sure it is adjusted to pH 7.1
No cytopathic effect or fluorescent signal following infection	Depending on the virus glycoprotein, cell lines other than HEK 293T or BHK‐21 can be required	VSV‐G has a very broad tropism; however, if VSV is used to select mutants, e.g., with antisera against glycoproteins of different viruses, the cell line must express the receptor for that glycoprotein; this can be achieved by choosing a cell line that naturally expresses the receptor or by stably introducing the receptor via lentiviral transduction
Virus rescue or production is unsuccessful	Fetal bovine serum containing VSV antibodies	Although unlikely, fetal bovine serum may contain antibodies against VSV, as cattle are one of the host species of VSV; VSV is primarily epidemic in the USA, therefore, using serum produced from outside the USA can fix the issue

### Understanding Results

When generating a new transgenic VSV to study the forced evolution of its transgene (e.g., SARS‐CoV‐2 M^pro^), the first assessment to be made is the extent of attenuation of the virus (see Table 6 and Table 7 for supplemental files and sequences). A gold‐standard technique in virology to purify and assess attenuation is the plaque assay (Fig. 3). Plaque size in correlation with time will reveal the replicative capacity of a newly generated virus. The addition of a transgene into the viral genome will likely result in an attenuation compared to the parental virus (without transgene). After selection of mutated transgene variants by performing the methodology outlined in Basic Protocol, it is important to perform dose response experiments to compare wild‐type and mutant variants.

**Table 6:** Supplemental Files*^a^*

File	Name	Description
Supplemental file 1	Assay calculation sheet	An Excel sheet to facilitate calculating amounts of cells, virus, and inhibitor in the assays
Supplemental file 2	Virus titration sheet	A printable template for counting infected and non‐infected wells as well as a formula sheet to calculate viral titers according to the method of Spearman and Kärber
Supplemental file 3	Sequencing and raw data	An Excel sheet containing an exemplary data set of a VSV mutant selection experiment, and the raw data used for making figures in this protocol

^*a*^ Supplemental files 1 and 2 are calculation aids; Supplemental file 3 contains raw data used to compile graphs and an exemplary collection of sequences mutation. Supplemental files 1 and 2 were adapted with permission from Rauch et al. (2024).

**Table 7:** Supplemental Sequences^*a*^

File	Name	Description
Supplemental sequence 1	VSV‐M^pro^	VSV with replacement of intergenic region between G and L with SARS‐CoV‐2 M^pro^
Supplemental sequence 2	VSV‐MERS‐M^pro^	VSV with replacement of intergenic region between G and L with MERS‐CoV M^pro^
Supplemental sequence 3	VSV‐M^pro^‐PmNeon	VSV with replacement of intergenic region between G and L with SARS‐CoV‐2 M^pro^ and intramolecular insertion of mNeon into P
Supplemental sequence 4	T7 polymerase	Phage T7 polymerase in lentiviral backbone with blasticidin resistance
Supplemental sequence 5	VSV‐N	VSV nucleoprotein gene in lentiviral backbone with puromycin resistance
Supplemental sequence 6	VSV‐P	VSV phosphoprotein gene in lentiviral backbone with hygromycin resistance
Supplemental sequence 7	VSV‐L	VSV polymerase gene in lentiviral backbone with blasticidin resistance

^*a*^ The plasmids necessary to carry out the described protocols are further detailed here. The sequences are provided as supplemental files in the GenBank (.gb) format, a standard format that includes sequence annotations.

**Figure 6:**
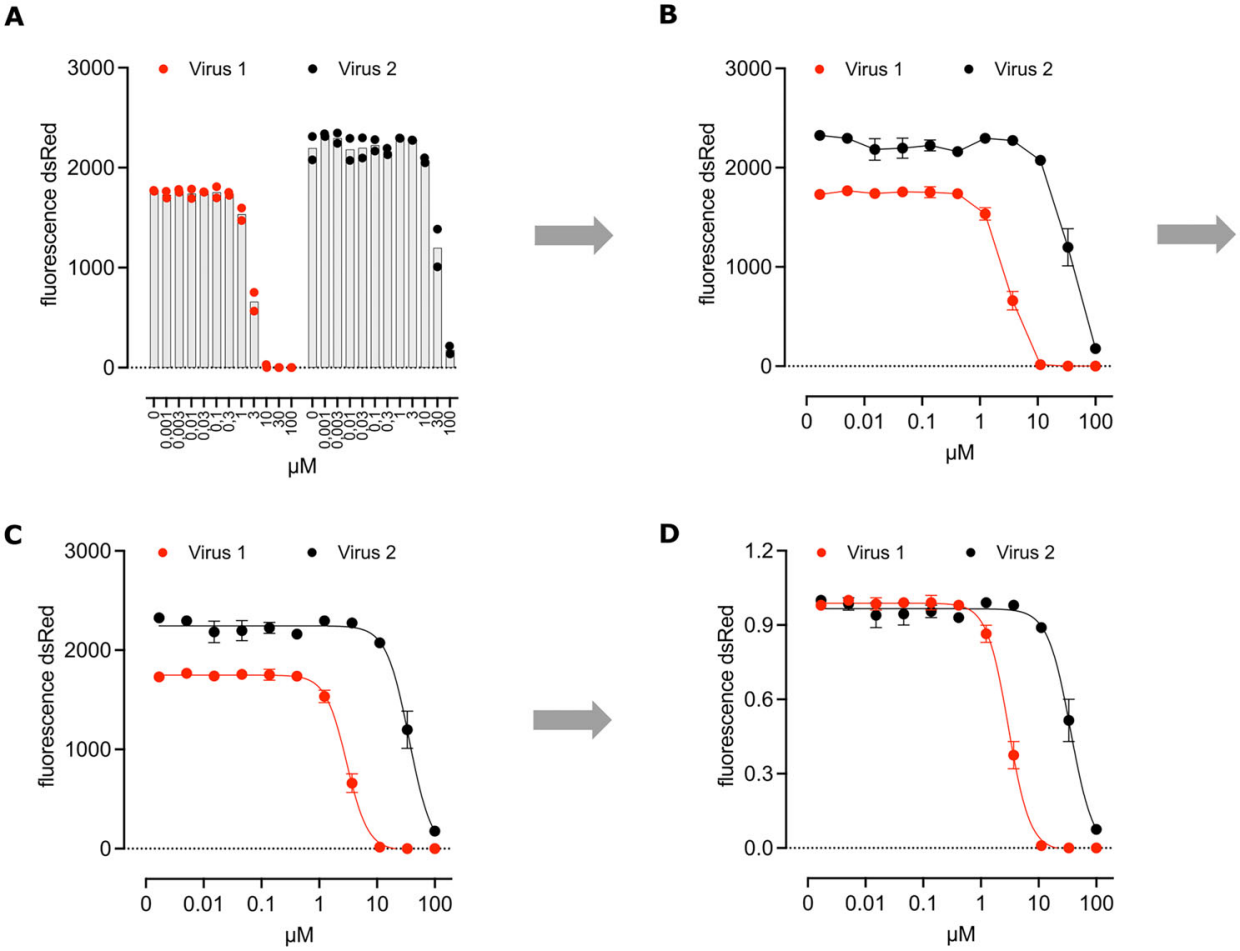
Exemplary data. Dose response curve of wild‐type VSV and a mutant transgenic VSV variant against the same inhibitor displayed as column (**A**), line (**B**), curve fit (**C**) and normalized graph (**D**).

The optimized form of this protocol uses VSV expressing a fluorescent protein to facilitate readouts. One graph type to display resulting dose responses visualized by the fluorescent signal are column diagrams (Fig. 6A). From these diagrams, a shift in the response can be assessed by determining at which concentration on the x‐axis the fluorescence starts fading and vanishes entirely. A display option that further enhances comparability are line graphs, where the responses are not displayed next to each other, but together in one graph (Fig. 6B). To precisely quantify the response, it is useful to calculate IC_50_ values, i.e., the dose at which 50% of the viral replication is inhibited. This value will differ from wild‐type to resistant mutants. In programs such as GraphPad Prism, IC_50_ values can be calculated by curve fitting, e.g., with a non‐linear model as described in our protocol (Fig. 6C). The maximum fluorescent signal emitted by the replicating virus can be different between different mutant variants, especially when this phenomenon has nothing to do with the resistance, but rather a mutation has an impact on viral replication. For example, the total fluorescent spot count (collected via ELISpot/FluoroSpot) of one mutant is higher than that of a different mutant. Therefore, plotting absolute spot count from dose response data could confuse the reader, as one curve would plateau higher than another. Normalizing each dataset (each mutant virus) by the highest mean spot count value improves the visual comparability and clarity, resulting in overlapping plateaus of fluorescent signals of the different viral variants. Dose response curves will only differ depending on the resistance shift against the applied inhibitor (Fig. 6D).

### Time Considerations

The two most time intense steps in this protocol are the generation of transgenic VSV, and its “rescue” from the plasmid encoding the viral genome. This method facilitates producing VSV (negative, single‐stranded RNA) particles starting from plasmid DNA. When rescuing standard VSV, replicating viral particles are produced within a few days post transfection. However, when the virus is attenuated, a rescue can take several weeks to months because the resulting virus must acquire secondary mutations to accommodate the new transgene (Heilmann et al., 2019). In some cases, transgenic VSVs become temperature sensitive (Printz & Wagner, 1971). This is not an issue per se as passaging with a selection pressure can also be done at lower temperatures (e.g., 35°C instead of 37°C). It is therefore sensible to try different temperatures if the rescue fails initially, or if the virus stops replicating once transferred to cells not transfected with the helper plasmids.

The number of required serial passages will depend on factors such as the viral replication kinetics and the intended repetition of the passage. Standard VSV can be passaged daily, as completing the replication cycle only takes several hours (Holzwarth et al., 2020). However, for a slower replicating, attenuated transgenic VSV, 2 to 3 days should be considered per passage. Furthermore, 10 passages may be required to select for mutant virus, which can take up to 1 month.

### Author Contributions

**Francesco Costacurta**: Methodology; visualization; writing—original draft; writing—review and editing. **Stefanie Rauch**: Investigation; methodology; visualization. **Dorothee von Laer**: Resources; supervision. **Emmanuel Heilmann**: Conceptualization; funding acquisition; investigation; methodology; project administration; supervision; validation; visualization; writing—original draft; writing—review and editing.

### Conflict of Interest

The authors declare no conflict of interest.

## Supporting information




*Figure S1. Suboptimal and optimal cell densities for plaque purification*.

*Supplemental file 1: Assay calculation sheet (see Table 6)*.

*Supplemental file 2: Virus titration sheet (see Table 6)*.

*Supplemental file 3: Sequencing and raw data (see Table 6)*.

*Supplemental sequence 1: VSV‐M^pro^ (see Table 7)*.

*Supplemental sequence 2: VSV‐MERS‐M^pro^ (see Table 7)*.

*Supplemental sequence 3: VSV‐M^pro^‐PmNeon (see Table 7)*.

*Supplemental sequence 4: T7 polymerase (see Table 7)*.

*Supplemental sequence 5: VSV‐N (see Table 7)*.

*Supplemental sequence 6: VSV‐P (see Table 7)*.

*Supplemental sequence 7: VSV‐L (see Table 7)*.

## Data Availability

All data provided for this protocol is summarized in a raw date file (see Supporting Information, Supplemental file 3).
