# Supplementary material for: Using Vesicular Stomatitis Virus as a Platform for Directed Protease Evolution
Source: Curr Protoc. 2024 Dec 23;4(12):e70074. doi: 10.1002/cpz1.70074 (PMC11664493; doi:10.1002/cpz1.70074)
Supplement: Supplementary file 1 — Figure S1. Suboptimal and optimal cell densities for plaque purification. [file CPZ1-4-0-s008.pdf]

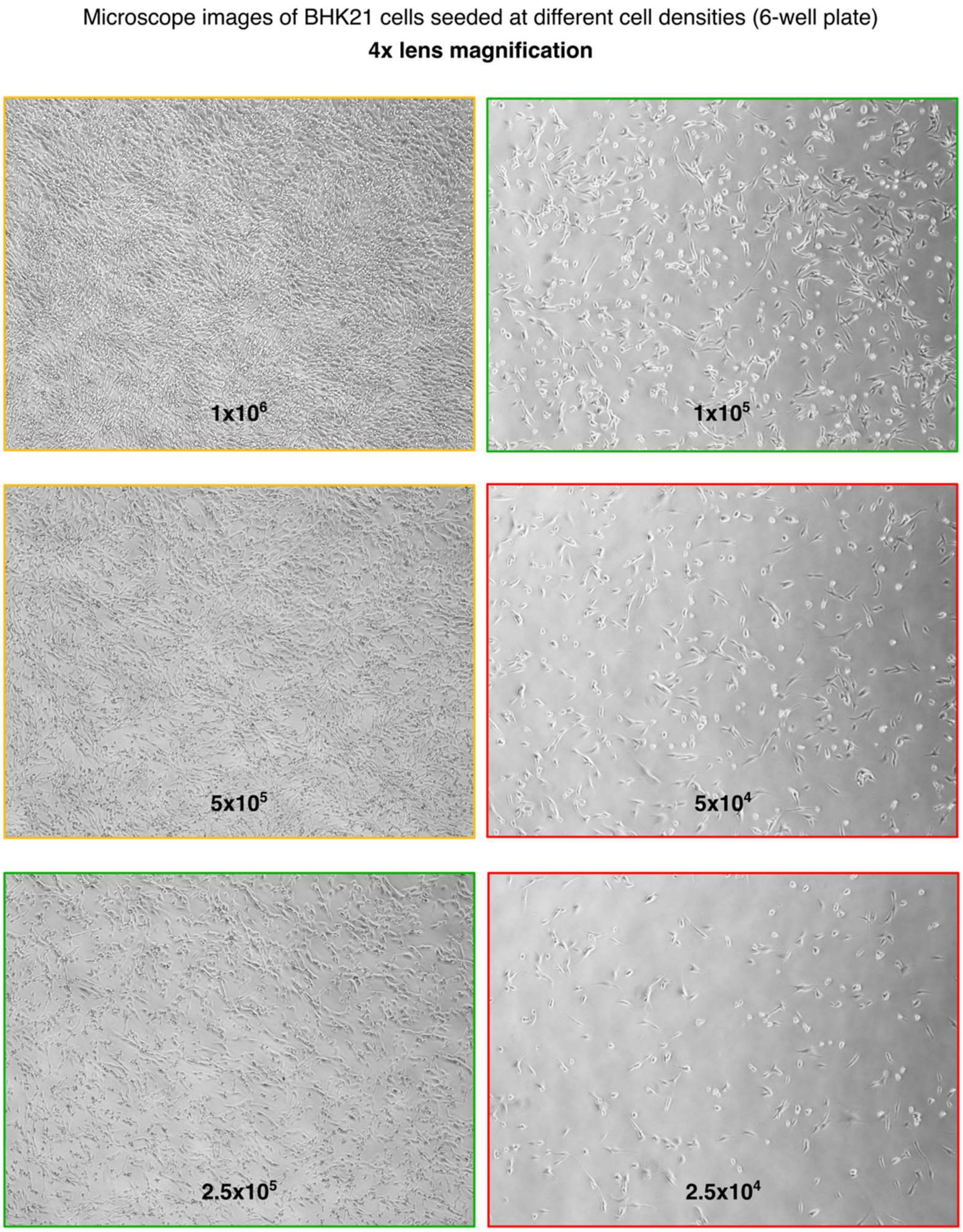

Figure S1. Suboptimal and optimal cell densities for plaque purification. BHK21 cells were seeded one day prior to infection. Yellow-orange outlined images represent very dense cell layers that can be used for plaque purification of fast replicating viruses (i.e. VSV). Green contoured images represent optimal cell densities for viruses that are attenuated (i.e. chimeric VSVs). Red outlined images represent low density cell layers that are not optimal for plaque purification, as plaques would easily be confused with areas where cells are not present.
